# Supplementary material for: MetaRibo-Seq measures translation in microbiomes
Source: Nat Commun. 2020 Jun 29;11:3268. doi: 10.1038/s41467-020-17081-z (PMC7324362; doi:10.1038/s41467-020-17081-z)
Supplement: Supplementary file 10 — Supplementary Data 7 [file 41467_2020_17081_MOESM10_ESM.zip › File2/Confidence_VeryHigh_Taxonomy/10417_out.krona.html]

Javascript must be enabled to view this page.

members
magnitude
magnitudeUnassigned
count
unassigned
taxon
rank

10417\_out


SRS1041145\_contig\_number\_31941
6
1

superkingdom
2759
1

1
4751
kingdom

subkingdom
1
451864

phylum
1
5204

subphylum
29000
1

class
162481
1

order
1
231213

1799696
1
family

genus
1
5533


SRS098571\_contig\_number\_66059
1
5286
species


SRS019685\_contig\_number\_8610SRS053356\_contig\_number\_contig-100\_1860.214294
2

2
2
superkingdom

phylum
201174
1

1760
1
class

order
1
85006


SRS051031\_contig\_number\_21030
1
2026762
species

1224
1
phylum

28211
1
class

1
204455
order

31989
1
family

genus
1
1649481

337702
1

SRS023914\_contig\_number\_24918
species
